# Dobzhansky-Muller and *Wolbachia*-Induced Incompatibilities in a Diploid Genetic System

**DOI:** 10.1371/journal.pone.0095488

**Published:** 2014-04-23

**Authors:** Arndt Telschow, Kirsten Hilgenboecker, Peter Hammerstein, John H. Werren

**Affiliations:** 1 Center for Ecological Research, Kyoto University, Otsu, Shiga, Japan; 2 Institute for Evolution and Biodiversity, Westfalian Wilhelms-University Münster, Münster, Germany; 3 Institute for Theoretical Biology, Humboldt University Berlin, Berlin, Germany; 4 Department of Biology, University of Rochester, Rochester, New York, United States of America; University of Poitiers, France

## Abstract

Genetic incompatibilities are supposed to play an important role in speciation. A general (theoretical) problem is to explain the persistence of genetic diversity after secondary contact. Previous theoretical work has pointed out that Dobzhansky-Muller incompatibilities (DMI) are not stable in the face of migration unless local selection acts on the alleles involved in incompatibility. With local selection, genetic variability exists up to a critical migration rate but is lost when migration exceeds this threshold value. Here, we investigate the effect of intracellular bacteria *Wolbachia* on the stability of hybrid zones formed after the Dobzhansky Muller model. *Wolbachia* are known to cause a cytoplasmic incompatibility (CI) within and between species. Incorporating intracellular bacteria *Wolbachia* can lead to a significant increase of critical migration rates and maintenance of divergence, primarily because *Wolbachia*-induced incompatibility acts to reduce frequencies of F1 hybrids. *Wolbachia* infect up to two-thirds of all insect species and it is therefore likely that CI co-occurs with DMI in nature. The results indicate that both isolating mechanisms strengthen each other and under some circumstances act synergistically. Thus they can drive speciation processes more forcefully than either when acting alone.

## Introduction

The Dobzhansky-Muller model has been used to study speciation in a variety of contexts [1]–[4]. The original model [5]–[6] considered individuals with two alleles at each of two loci (ancestral population with genotype *aabb*). When this population becomes separated into two geographically isolated parts, mutant alleles *A* and *B* occur in each subpopulation respectively and replace the ancestral allele. Thereby, the model makes no assumptions about how new alleles have been evolved, whether by drift or natural selection. When populations reconnect after periods of isolation in the so-called secondary contact, the occurrence of *A* and *B* in one individual can lead to hybrid dysfunctions. An important point of the Dobzhansky-Muller model is that neither *A* or *B* have deleterious effects in their own subpopulation's genetic background nor are they deleterious by themselves. However, they might cause hybrid dysfunctions in the presence of each other [2], [5], [7]. Thus, reproductive isolation evolves as a side-effect of evolution in allopatry.

Dobzhansky-type epistatic models require that viable or optimal genotypes are connected by a viable intermediate genotype [1]–[3]. *AAbb* can evolve from *aabb* through *Aabb* without passing through any deep adaptive valley. Individuals *aabb*, *Aabb* and *AAbb* are neither reproductively isolated nor do they suffer from sterility or inviability. This applies likewise to the group of genotypes *aabb*, *aaBb* and *aaBB*. The original Dobzhansky-Muller (DM) model only considers two alleles that cause hybrid dysfunctions, but it can also be assumed that there are many of these incompatible pairs which all have small effect on fitness [8]. Examples of single loci with large effects on hybrid sterility or inviability are known [8]–[11]. Regarding the stability of Dobzhansky-type incompatibilities in parapatric populations, i.e. the maintenance of all four alleles, Gavrilets (1997) stated that genetic diversity is not maintained if there is migration between the two populations [3]. Recent theoretical studies indicate that stability can be achieved when DM incompatibility alleles are also subject to local selection. Then, maintenance of diversity is possible up to a threshold migration rate that is basically determined by the strength of local selection and the degree of nuclear incompatibilities [12], [13].

Besides nuclear-based factors, it is further possible that cytoplasmic endosymbionts can influence speciation processes of their hosts. Prominent representatives of such organisms are *Wolbachia* bacteria, and there is strong evidence that they have impact on evolution of their hosts [14]–[17]. *Wolbachia* are among the most common endosymbionts in the world, with 20–70% insect species estimated to be infected [18]–[20]. *Wolbachia* can manipulate the reproduction of their hosts by inducing a cytoplasmic mating incompatibility or CI (for reviews see [21]–[23]). CI involves a sperm-egg incompatibility: *Wolbachia* induce modification of sperm and rescue in infected eggs. Disruption of sperm chromosomes occurs when the sperm is modified, but the same strain is not present in the egg to rescue. Unidirectional CI typically occurs when the male is infected but the female is not, whereas bidirectional CI occurs when both mating partners are infected with strains that have different mod-rescue systems [21].

CI has received attention as a possible isolating mechanism dating from its discovery [14] onwards (e.g. [15]–[17], [24]). Both theoretical [25], [26] and empirical [15], [17], [27], [28] studies provide evidence for *Wolbachia* playing a role in speciation processes of their hosts; however the view remains controversial (e.g. [29], [30]). A major concern is that *Wolbachia* induced incompatibilities are insufficient for promoting speciation in the face of migration, particularly when cytoplasmic incompatibility is unidirectional. Recent theoretical treatments have shown that both uni- and bidirectional CI can strongly reduce gene flow and select for premating isolation between parapatric host populations, if migration is below a critical value [25], [26], [31], [32]. The critical migration rates can reach high values for bidirectional CI, but are predicted to be low for unidirectional CI [33], [34]. However, most of these models use a haploid genetic system (but see [32]), which is convenient for modeling purposes but lacks biological realism for most eukaryotes. Diploid genetics is especially important in models of speciation as recessive nuclear incompatibilities are thought to play a crucial role in early stages [30], [35], [36].

As pointed out by several authors [16], [37], [38], multiple isolating mechanisms in combination are likely to be involved in reproductive isolation, even in the early stages. Due to the abundance of *Wolbachia* among insects and other arthropods, CI may frequently co-occur with nuclear incompatibilities during evolutionary processes. Indeed, such cases have been found in *Nasonia* [15], *Drosophila* [27] and also spider mites [39].

In the present study we investigate the effects of *Wolbachia* on the stability of Dobzhansky-Muller incompatibilities in a diploid genetic system, allowing for variable levels of dominance. First, we consider DMI without CI, and show that nuclear incompatibility allele diversity only persists when the alleles are also under local selection, and if migration is sufficiently low or dominance in incompatibility high (*Scenario A*). These results confirm previous theoretical work [12], [13], and serve as a baseline for the models with *Wolbachia*. Second, we demonstrate that in the presence of *Wolbachia* induced CI, the stability of genetic divergence increases significantly, particularly for recessive nuclear incompatibilities. This is true for the three scenarios considered that include either uni- or bidirectional CI (*Scenarios B*–*D*).

In general, these results suggest that a high degree of reproductive isolation as well as local positive selection on incompatibility alleles lead to a higher stability of hybrid zones and that the stabilizing effect of *Wolbachia* increases with increasing strength of CI. Our results further substantiate that *Wolbachia* might influence the evolution of their hosts. In addition to inducing a high degree of reproductive isolation between hosts infected with different strains, they select for the maintenance of nuclear incompatibilities.

## The Model

**Figure 1:**
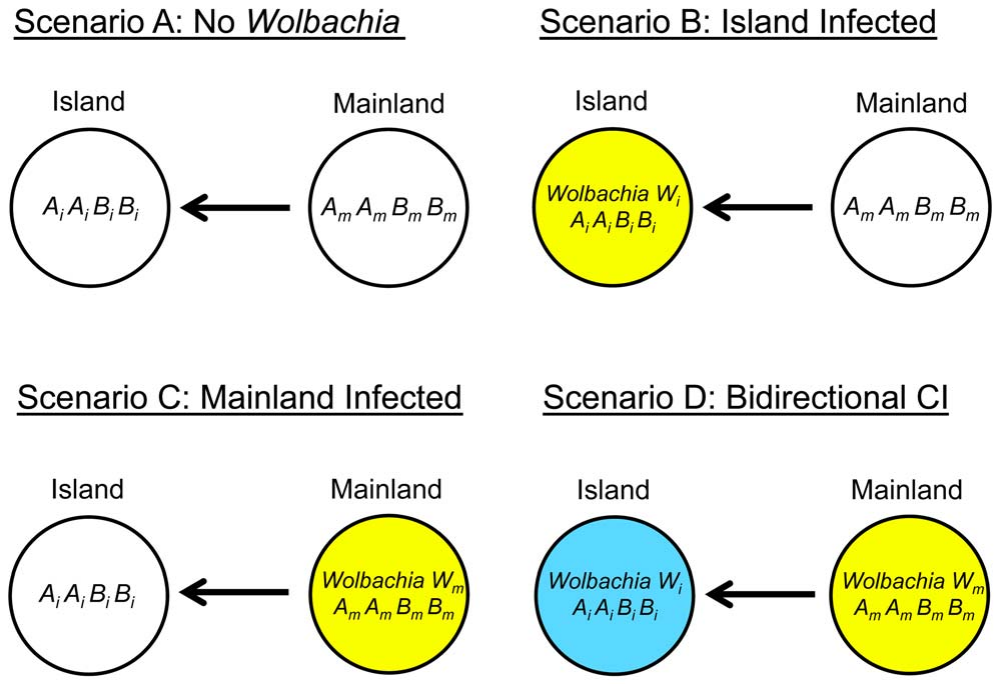
Basic model structures. *Scenario A*: The island population initially consists of individuals of genotype *A_i_ A_i_ B_i_ B_i_*, whereas the mainland is homogeneous for genotype *A_m_A_m_B_m_B_m_*. Local selection acts on allele *A_i_* on the island. After secondary contact is restored, genetic incompatibilities can occur between *A_i_* and *B_m_*. The model is extended by including *Wolbachia* in scenarios *B*–*D*. Colors indicate uninfected (white) and *Wolbachia*-infected populations (yellow and blue). *Scenario B*: Island infected with *Wolbachia* and mainland uninfected. *Scenario C*: Mainland infected with *Wolbachia* and no infection on the island before secondary contact. *Scenario D*: Before secondary contact, the island is infected with *Wolbachia* strain *W_i_* and the mainland population with strain *W_m_*. Scenarios *B* and *C* involve unidirectional CI, whereas scenario *D* involves bidirectional CI. After secondary contact, both nuclear and *Wolbachia*-induced cytoplasmic incompatibilities reduce number of offspring in intergroup matings.

In this section we present a verbal description of the model. A mathematical formalization is provided in Appendix S1. The basic structure uses diploid genetics, and is as follows: Two populations (a mainland and an island population) have diverged at nuclear loci and (possibly) regarding their *Wolbachia* strains. The populations are now in secondary contact (Figure 1), with migration from the mainland to the island. We consider the dynamics of two nuclear incompatibility loci (*A* and *B*), which are also subject to local selection pressures in the two populations (e.g., allele *A_i_* is favored in the island population and allele *B_m_* is favored in the mainland population). Initially, the two populations are fixed for their alternate alleles (*B_i_* in the island and *A_m_* in the mainland). We assume that in combination alleles *A_i_* and *B_m_* show Dobzhansky-Muller incompatibilities, with the dominance of the incompatibility interaction being variable “*h*” (Table 1). In addition, the populations may also have diverged in their *Wolbachia* CI-types, such that uni- or bidirectional incompatibility occurs between them at level *l_CI_* (Table 2). We explore the dynamics on the island either with or without the *Wolbachia* incompatibilities and as a function of dominance of the nuclear incompatibilities and strength of local selection.

**Table 1:** Dobzhansky-Muller incompatibilities.

	*A_m_A_m_*	*A_i_A_m_*	*A_i_A_i_*
*B_i_B_i_*	1	1	1
*B_i_B_m_*	1	1-*hl*_NI_	1-*hl*_NI_
*B_m_B_m_*	1	1-*hl*_NI_	1-*l*_NI_

**Table 2:** *Wolbachia*-induced cytoplasmic incompatibility (CI).

Bidirectional CI			Unidirectional CI		
	male			male	
Female	*W*_i_	*W*_m_	Female	*W*	*O*
*W*_i_	1	1-*l*_CI_	*W*	1	1
*W*_m_	1-*l*_CI_	1	*O*	1-*l*_CI_	1

The genotype is characterized by two alleles at each of two loci and the cytotype denotes the individuals' *Wolbachia* infection status. Before secondary contact starts, the island population consists of individuals of genotype *A_i_A_i_B_i_B_i_*, whereas organisms of the mainland population are of genotype *A_m_A_m_B_m_B_m_*. We first consider the dynamics of the locally adapted hybrid incompatibility alleles (*Scenario A*). We then add in cytoplasmic incompatibility by *Wolbachia*, utilizing scenarios with either uni- or bidirectional CI. Unidirectional CI involves one *Wolbachia* strain. In *Scenarios B*, we consider the situation with an uninfected mainland, and in *Scenario C* with an infected mainland. Bidirectional CI involves two *Wolbachia* strains, and the populations have different infections before secondary contact (*Scenario D*).

The individuals' lifecycle consists of three steps, migration, local viability selection and then reproduction. Individuals are diploid at all non-gametic stages. For all scenarios, we consider a mainland island structure with one-way migration. At each time step, a fraction *m* of the island population is replaced by individuals of the mainland population. This fraction is called the migration rate. After migration, viability selection takes place on the locally adapted alleles. We assume that allele *A_i_* is positively selected in the island population. Individuals with genotype *A_i_A_m_* and *A_i_A_i_* have a 1+*s*/2 and 1+*s* times higher probability to survive than *A_m_A_m_* individuals, making the local fitness effect co-dominant.

In the reproduction step, we consider the inheritance of the alleles, the transmission of *Wolbachia* as well as nuclear and cytoplasmic incompatibilities. We assume that the nuclear loci are unlinked, resulting in complete recombination between them. *Wolbachia* infection is maternally transmitted and we assume perfect transmission of *Wolbachia*, i.e. all offspring carry the same strain as their mother. In the model with unidirectional CI, we further assume that infected females have a (1-*f*) times lower fecundity than uninfected females [34], [40].

By modeling nuclear incompatibilities (NI), we follow a model by Turelli and Orr [41]. It assumes that the degree of fitness reduction in one individual depends on its number of incompatible alleles. NI occurs between the *A* and *B* locus as described in Table 1. Thereby, we call *l*_NI_ the NI level and *h* the dominance factor. We allow *h* to vary from completely recessive (*h* = 0) to completely dominant (*h* = 1) incompatibility. Cytoplasmic incompatibility occurs in matings where the male is infected and the female either uninfected (unidirectional CI) or infected with a different strain of *Wolbachia* (bidirectional CI). The proportion of inviable offspring is defined as the CI level, *l*_CI_ (Table 2). Accordingly, incompatible crosses produce a 1-*l*_CI_ proportion lower number of viable offspring relative to compatible crosses.

In general, the dynamics of this diploid model cannot be studied analytically (although we provide some analytical approximations below). Therefore, most results presented are obtained from numerical analysis. Our investigations mainly focus on finding parameter ranges that allow stable coexistence of all four alleles on the island. Therefore, equilibrium frequencies of alleles have to be determined. To do so, we defined a termination condition and ended numerical iterations if at least 10^5^ time generations have past and the sum of the differences of frequencies of the single geno- and cytotypes from one generation to the next was less than 10^−10^. We then perturbed the frequencies above and below the achieved frequencies and continued the iterations until the termination condition was reached again. These frequencies are called equilibrium frequencies. We say that a certain geno- or cytotype has become fixed in a population if its frequency has exceeded 99.9%. Critical migration rates are determined with an accuracy of 10^−6^.

## Results

### Scenario A: Dobzhansky-Muller Model without CI

We aim at investigating the effect of *Wolbachia* on nuclear incompatibilities. Before elaborating on the dynamics of this extended model, we first analyze the Dobzhansky-Muller model in the absence of CI-bacteria and examine the conditions under which genetic diversity is maintained (i.e., when all four alleles coexist in the island population).

Before secondary contact is established, the island population consists of individuals of genotype *A_i_A_i_B_i_B_i_*. On the mainland, individuals can be characterized by their genotype *A_m_A_m_B_m_B_m_*. Since there is no migration onto the mainland, frequencies remain constant. With the beginning of secondary contact, the island population receives migrants from the mainland. Whether resident alleles can coexist with migrant alleles *A_m_* and *B_m_* or *A_i_* and *B_i_* become extinct depends on the migration rate (*m*), the selection coefficient (*s*), and on the degree and architecture of reproductive isolation (*l*_NI_, *h*).

**Figure 2:**
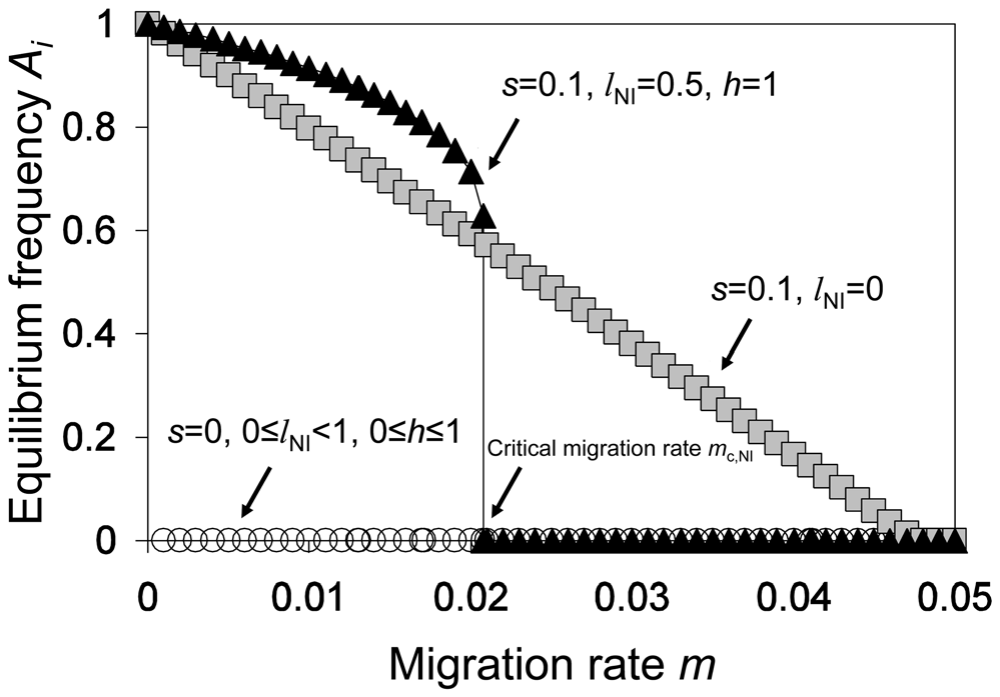
Equilibrium frequencies of allele *A_i_* on the island population as a function of the migration rate. There are three qualitatively different outcomes. Circles show a typical example for the case without local selection (*s* = 0), boxes for the case with local selection (*s*>0) but without nuclear incompatibilities (*l*_NI_ = 0), and triangles for the case in which both local selection (*s*>0) and nuclear incompatibilities (0<*l*_NI_≤1) act. The critical migration rate of nuclear divergence (*m*_c,NI_) is indicated by an arrow.

Let us first consider the model without local selection. In accordance with Gavrilets [3], we found that as long as reproductive isolation is imperfect, *A_m_* and *B_m_* spread on the island while *A_i_* and *B_i_* go to extinction. Although there is an isolating barrier against the *B_m_* allele, *A_m_* is not affected by hybrid incompatibilities and replaces *A_i_*. Thereafter, since *B_m_* is only deleterious co-occurring with *A_i_*, the isolating barrier is lost and *B_m_* goes to fixation as well. Let us now consider the model with local selection but without nuclear incompatibilities. Figure 2 shows that the equilibrium frequency of *A_i_* decreases continuously with increasing migration rate. Allele *A_i_* can persist in the presence of sufficiently low migration because it is favored by local selection. In contrast, *B_i_* becomes extinct for any value of the migration rate. In conclusion, if either local selection or nuclear incompatibilities act separately, there is no stable coexistence of all four alleles.

We next consider the joint action of Dobzhansky-Muller incompatibilities and local selection. In concordance with Bank *et al.* [12] and Wang [13], we find that local selection is necessary for the maintenance of Dobzhansky-Muller incompatibilities, except for the special case of complete dominance and maximal nuclear incompatibility (*l*_NI_ = 1 and *h* = 1). Figure 2 shows equilibrium frequencies of *A_i_* for the case where incompatibility dominance is 1, but lethality is ½. Below a certain value of the migration rate, *A_i_* and *A_m_* coexist on the island. The same is true for alleles at the B-locus. When migration exceeds this critical value, both island alleles *A_i_* and *B_i_* go to extinction, despite the *A_i_* allele being locally adapted. This critical value will be referred to as the *critical migration rate of nuclear divergence* (*m*_c,NI_). If migration is below the critical value, all four alleles stably coexist; if migration exceeds the threshold value, resident alleles go to extinction.

**Figure 3:**
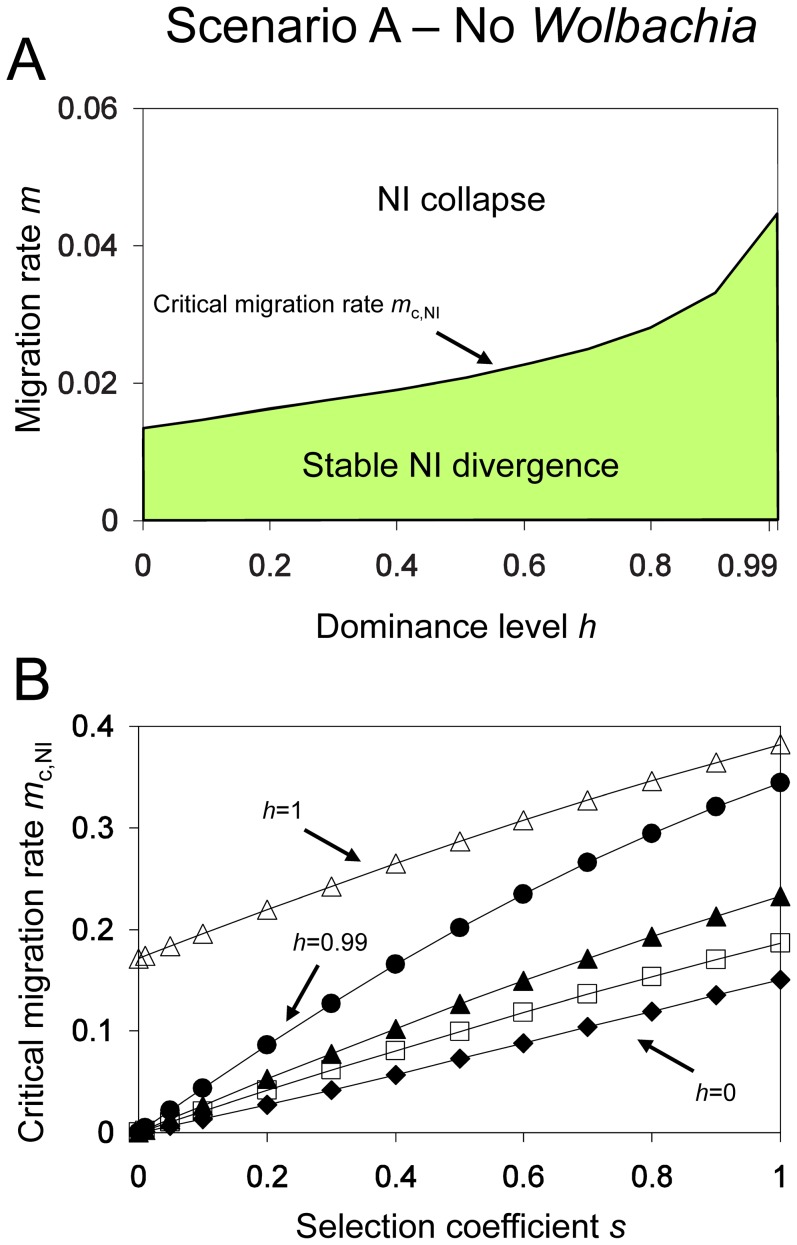
Stability of Dobzhansky-Muller incompatibilities (DMI) for *Scenario A* (No *Wolbachia*). *Graph A*: The critical migration rate (*m*_c,NI_) divides the parameter plane spanned by dominance level (*h*) and migration rate (*m*) into two regions. If *m*<*m*_c,NI_ then alleles *A_i_* and *B_i_* can persist on the island, and genetic divergence at both loci is stable (green region). For all other parameter constellations, polymorphism at either locus is lost and DMI disappear (white region). The graph further shows that stability of genetic divergence increases with degree of genetic incompatibility. Parameters: *l*_NI_ = 1 and *s* = 0.1. *Graph B*: Critical migration rates as functions of the selection coefficient for different dominance levels *h* = 0 (diamonds), *h* = 0.5 (boxes), *h* = 0.75 (black triangles), *h* = 0.99 (circles) and *h* = 1 (white triangles). Local selection has increasing effects on stability with increasing selection coefficient, and the largest changes occur for nearly dominant hybrid lethality (*h* = 0.99). Other parameter: *l*_NI_ = 1.

In general, *m*_c,NI_ increases with increasing strength of reproductive isolation. Figure 3A shows that the critical migration rate increases with increasing dominance level. In other words, dominant incompatibilities persist up to higher critical migration rates than codominant and recessive NI. Similar to the effect of dominance, critical migration rates increase with increasing NI level (results not shown). The selection coefficient, i.e. the strength of local selection for the locally adapted allele *A_i_*, also has a strong effect on stability of nuclear divergence (Figure 3B). Positive selection on island alleles leads to a higher persistence against migrants, and thus critical migration rates increase with increasing strength of selection.

Figure 3B contrasts the effect of local selection for different levels of dominance. There are two important insights. First, critical migration rates are lowest for recessive incompatibilities (*h* = 0). When *h* = 0, negative fitness consequences are diminished in heterozygotes. For recessive incompatibilities, all F_1_ hybrids are completely viable. Deleterious alleles are only expressed in some F_2_ generation individuals (those resulting from matings that yield *A_m_A_m_B_i_B_i_* or *A_i_A_i_B_m_B_m_* progeny) and the mainland alleles can thus easily spread through populations due to “protection” in heterozgotes. Second, there is a significant qualitative difference between complete dominant incompatibilities (*h* = 1) and almost complete dominance (*h* = 0.99) when NI causes complete lethality (*l*_NI_ = 1). In the former case, subpopulations produce no viable offspring in hybrid matings. Even without local selection, individuals of subpopulations stably coexist up to high critical migration rates of 17.2%. This changes for an almost complete dominance level of *h* = 0.99, where genetic divergence is not maintained without local selection even at very low migration rates. The incompatibility allele *B_m_* can simply leak into the island population in this case, producing matings among mainland compatible genotypes, and thus allowing their easy invasion of the island population. Eventually, this results in the loss of alleles *A_i_* and *B_i_* on the island. Local adaptation is extinguished.

### Scenario B: uninfected mainland and unidirectional CI

We now add intracellular bacteria *Wolbachia* and study their influence on the stability of nuclear divergence. In *Scenario B*, we consider one *Wolbachia* strain causing unidirectional CI, and a population structure with an uninfected mainland (fig. 1B). In a model neglecting diploid nuclear genetics, it was shown that *Wolbachia* persist on the island in the face of migration for a broad range of parameters [34]. Analogously to the case with nuclear incompatibilities, there exists a critical migration rate (*m*_c,CI_) below which cytoplasmic divergence can stably persist, but above which uninfected migrants spread, resulting in *Wolbachia* extinction on the island. This critical migration rate can be calculated analytically, and computes to *m*_c,CI_ = 0.25 *l*_CI_ in the absence of costs of infection. High CI levels thus result in high values of *m*_c,CI_, and the maximal cytoplasmic critical migration rate is at 25%. Fecundity reductions in infected females or incomplete *Wolbachia* transmission reduce the critical migration rate (*m*_c,CI_), but only slightly for realistic parameters [34].

**Figure 4:**
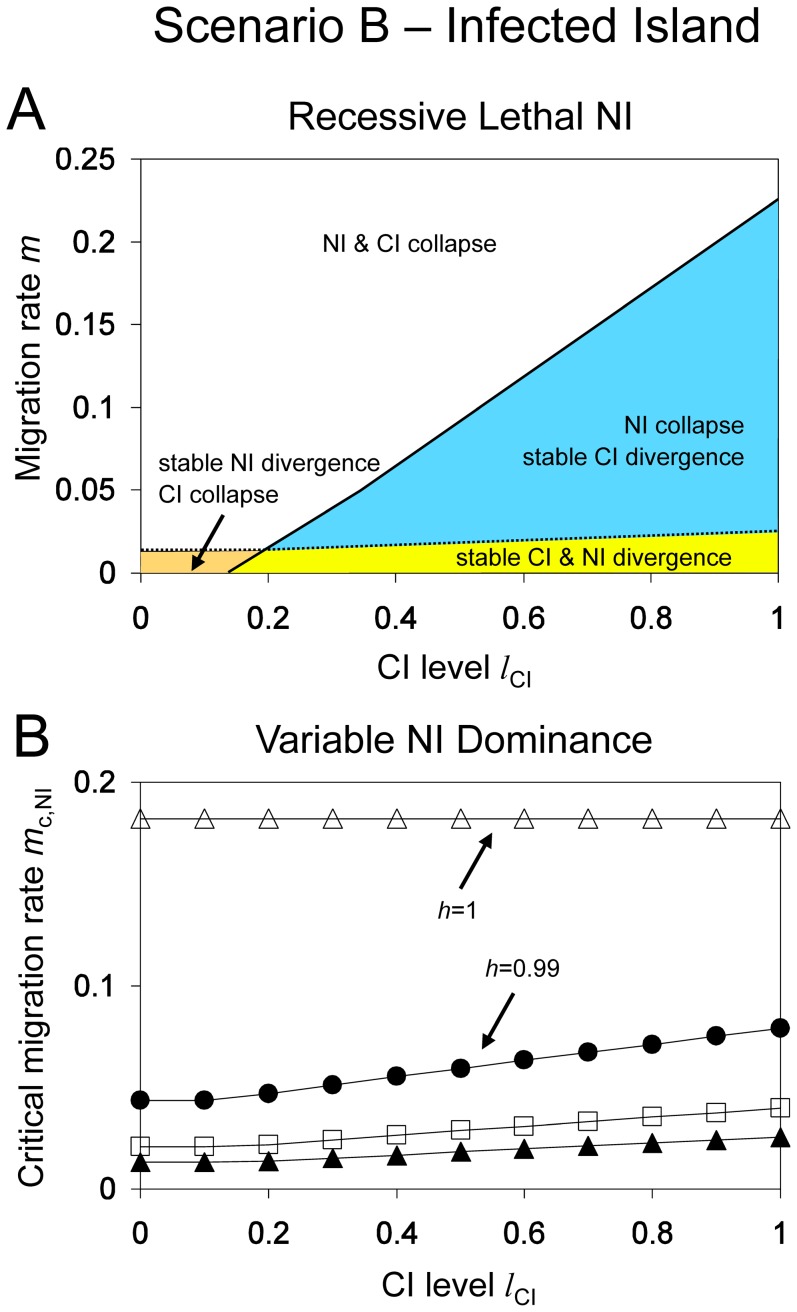
Stability of nuclear and cytoplasmic divergence for *Scenario B* (unidirectional CI with infected island population). *Graph A: Recessive Lethal NI*. The parameter plane is divided into four zones: (1) stable NI and loss of *Wolbachia* (orange), (2) stable NI and *Wolbachia* persistence (yellow), (3) loss of NI and *Wolbachia* persistence (blue), (4) loss of both NI and *Wolbachia* (white). The dotted and solid lines indicate the critical migration rates of NI and CI, respectively. Note that *Wolbachia* causes a fecundity reduction of *f* = 0.1. Other parameters: *l*_NI_ = 1, *h* = 0, *s* = 0.1. *Graph B: Variable Dominance NI*. Critical migration rates of nuclear divergence (*m*_c,NI_) as function of the CI level and for different dominance levels *h* = 0 (black triangles), *h* = 0.5 (boxes), *h* = 0.99 (circles) and *h* = 1 (empty triangles). Parameters: *l*_NI_ = 1, *s* = 0.1, *f* = 0.1.

Figure 4A illustrates how the two critical migration rates of nuclear (*m*_c,NI_) and cytoplasmic incompatibility (*m*_c,CI_) relate to each other. The graph shows that the parameter plane spanned by CI level and migration rate is divided into four zones: (1) stable NI and loss of *Wolbachia*, (2) stable NI and *Wolbachia* persistence, (3) loss of NI and *Wolbachia* persistence, and (4) loss of both NI and *Wolbachia*. For most parameter values, cytoplasmic incompatibility is more stable than nuclear incompatibility, i.e. *m*_c,CI_>*m*_c,NI_, and large differences occur for high CI levels. The opposite is true only for low CI levels of less than 20%.

To investigate the interactions of nuclear and cytoplasmic incompatibilities in more detail, we plotted the critical migration rates of NI as a function of the CI level. Figure 4B contrasts the effect of CI on the stability of NI for different dominance levels and an NI level of 1. When NI is completely dominant (*h* = 1), then the CI level has no effect on the critical migration rates for nuclear divergence. This is obvious – all hybrids die in the F1 generation even without CI. For all other dominance levels, however, presence of *Wolbachia* affects stability of NI. For example, the critical migration rate for recessive NI (*h* = 0) is 1.3% in the absence of *Wolbachia*, but increases to 1.8% for a CI level of 0.5, and to 2.4% for a CI level of 1. Similarly, the critical migration rate for nearly complete NI (*h* = 0.99) increases from 4.3% in the absence of *Wolbachia* to 7.8% for a CI level of 1. This demonstrates that CI can significantly stabilize NI, resulting in almost doubled critical migration rates for complete CI. We have also investigated whether NI stabilizes cytoplasmic divergence. This can indeed happen, but it occurs only for weak CI when critical migration rates of NI are higher than that of CI (results not shown).

### Scenario C: infected mainland and unidirectional CI

Next, we consider unidirectional CI with an infected mainland and an uninfected island (fig. 1C). Let us again first look at the case without local adaptation and nuclear incompatibilities. In general, *Wolbachia* infection easily spreads on the island upon secondary contact, and critical migration rates are either zero or close to it [34]. In contrast to *Scenario B*, the *Wolbachia* spread can only be prevented when there is a sufficient infection cost to infected females (e.g. when females are less fecund than uninfected females) or when maternal transmission of *Wolbachia* is imperfect. Nevertheless, critical migration rates are generally lower than for *Scenario B*. A further difference is that critical migration rates for *Wolbachia* invasion reach their maximum for intermediate CI levels.

**Figure 5:**
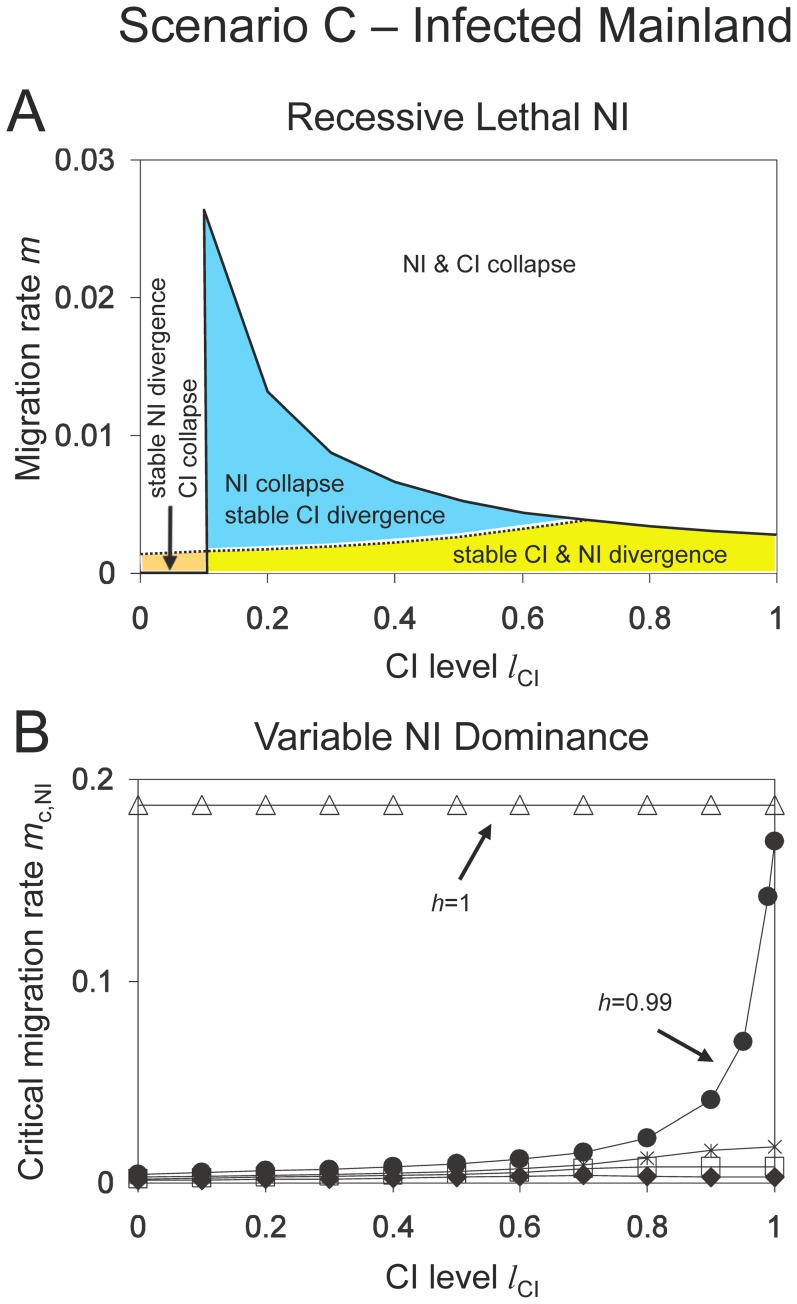
Stability of nuclear and cytoplasmic divergence for *Scenario C* (unidirectional CI with infected mainland population). *Graph A: Recessive Lethal NI.* Critical migration rates of NI (dotted line) and CI (solid line) as functions of the CI level. As in figure 4, the parameter space is divided into four zones. CI collapse is either caused by the spread of *Wolbachia* on the island (*l_CI_*>0.1) or by *Wolbachia* loss in both populations (*l_CI_*<0.1). Other parameters: *l*_NI_ = 1, *h* = 0, *s* = 0.01, *f* = 0.1. The graph shows that unidirectional CI has only weak effect on the stability of recessive NI. *Graph B: Variable Dominance NI.* Critical migration rates for nuclear divergence as functions of the CI level for different dominance levels *h* = 0 (diamonds), *h* = 0.5 (boxes), *h* = 0.75 (daggers), *h* = 0.99 (circles) and *h* = 1 (triangles). Parameters: *l*_NI_ = 1, *s* = 0.01, *f* = 0.1. Unidirectional CI has the largest effects on stability of NI when the CI level is high and dominance is near perfect, but has some leakage (e.g. *h*  = 0.99). In this case, CI and NI act synergistically in the sense that both CI and NI reach much higher critical migration rates than either alone in the corresponding mainland-island models.

Let us consider the full model including nuclear and cytoplasmic incompatibility, and local adaptation. As in *Scenario B*, there are four qualitatively different regions: (1) stable NI and loss of *Wolbachia*, (2) stable NI and *Wolbachia* persistence, (3) loss of NI and *Wolbachia* persistence, and (4) loss of both NI and *Wolbachia*. In contrast to *Scenario B*, there is a wide parameter range where unidirectional CI is maintained, but only up to smaller critical migration rates than genetic incompatibilities. Therefore, *Wolbachia* infection will not stabilize genetic divergence in these cases since cytoplasmic divergence collapses. This effect occurs for strong local selection pressures and/or weak cost of *Wolbachia* infection (results not shown). However, if local selection on nuclear alleles is weak (and therefore *m*_c,NI_ low), then *Wolbachia* increases stability of NI (fig. 5). The increase is largest for recessive nuclear incompatibilities because these are least stable against migration. Interestingly, the effect is most pronounced for intermediate CI because critical migration rates of *Wolbachia* are highest for these values (fig. 5A).

Figure 5B contrasts stability increases of nuclear divergence for different dominance levels. Threshold levels for recessive incompatibilities are moderately affected. Critical migration rates increase from 0.13% up to 0.27%, and the increase is from 0.21% to 0.79% for codominant NI. This is in the same order as in *Scenario B* (fig. 4B). For almost perfect NI (*h* = 0.99), however, critical migration rates increase from 0.44% up to 16.9% for full CI, showing a strong effect of *Wolbachia*. In this scenario, both isolating mechanisms interact synergistically so that both are significantly stabilized, in contrast to models considering only one isolating mechanism.

In order to explain the synergistic effect, let us consider the case with complete cytoplasmic incompatibility (*l*_CI_ = 1). Under this assumption, gene flow from an infected mainland to an uninfected island is reduced to zero [26], [31]. Accordingly, mainland alleles *A_m_* and *B*_m_ cannot spread on an uninfected island as long as *Wolbachia* is not spreading (note that this is in stark contrast to *Scenario B*). This implies that *m*_c,CI_≤*m*_c,NI_, and explains the stabilizing effect of CI on NI. In contrast, nuclear incompatibilities (which are stabilized by CI) can prevent the spread of *Wolbachia* and thus stabilize unidirectional CI. However, this happens only if the fitness of hybrid females is strongly reduced by NI. As the strongest fitness reduction occurs for strong NI and (nearly) complete dominance, the synergistic effect is most pronounced under these circumstances.

In conclusion, we find that unidirectional CI can enhance maintenance of Dobzhansky-Muller incompatibilities. In mainland-island models with an infected island (*Scenario B*), nuclear divergence is stabilized under a broad range of conditions, and the effect is strongest for high CI levels. Models with an infected mainland (*Scenario C*) show complex results. Stability increase of NI is most pronounced for weak local adaptation and intermediate CI levels. However, strong effects occur for cases with nearly complete dominance. Then, CI and NI act in synergy and reach high critical migration rates up to 17% for both isolating mechanisms.

### Scenario D: bidirectional CI

We now add a second strain of *Wolbachia* and study the influence of bidirectional CI on the stability of nuclear divergence (*Scenario D*). Bidirectional CI occurs when two *Wolbachia* strains are mutually incompatible [21]. The stability of coexistence was examined by Telschow *et al.* [33]. In a model neglecting nuclear genetics, the critical migration rate was calculated analytically and shown to increase with increasing CI level from 0 (*l*_CI_ = 0) up to 17% for perfect CI (*l*_CI_ = 1). If migration exceeds the critical migration rate, the migrant *Wolbachia* strain spreads on the island while the resident strain goes to extinction.

**Figure 6:**
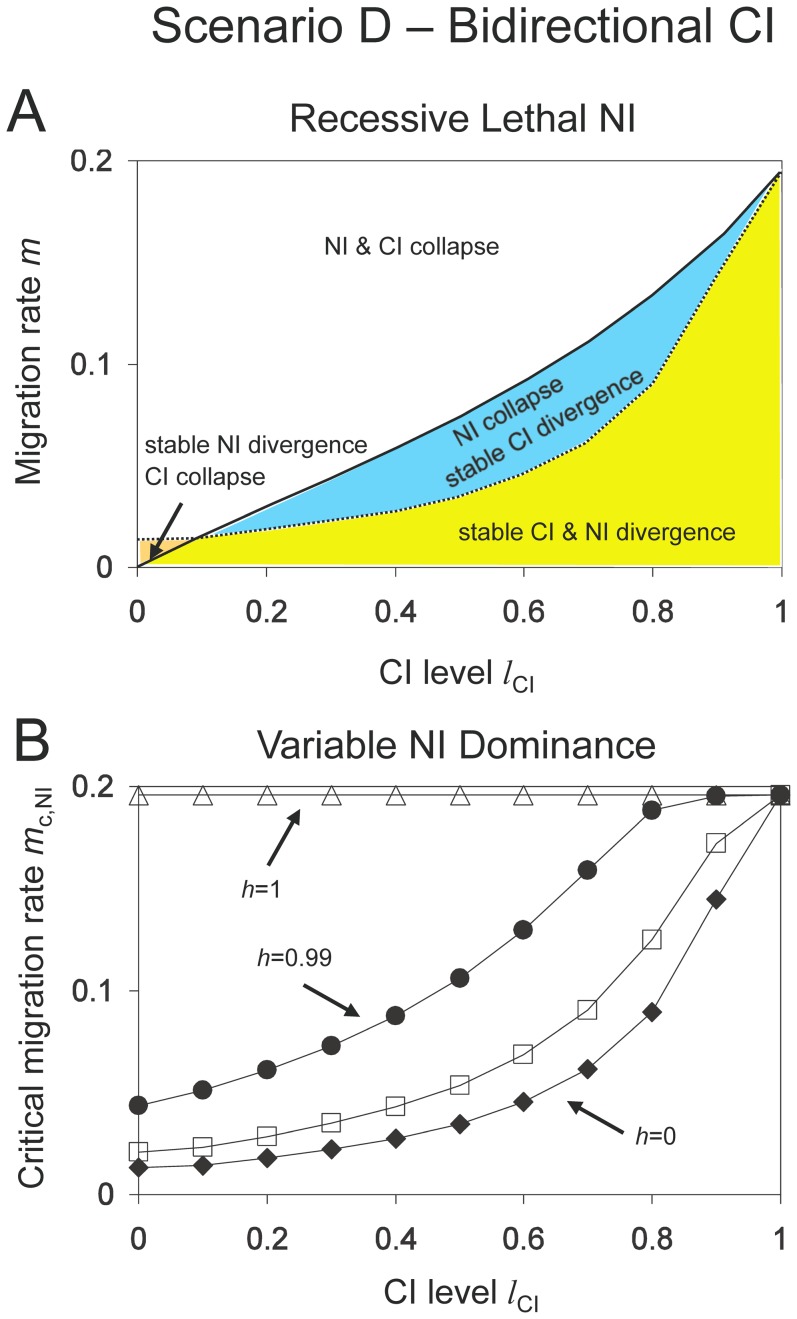
Stability of nuclear and cytoplasmic divergence for *Scenario D* (bidirectional CI). *Graph A: Recessive Lethal NI.* Bidirectional CI increases stability of nuclear divergence. The graph shows that the parameter plane is divided into four zones: (1) stable NI and loss bidirectional CI (orange), (2) stable NI and bidirectional CI (yellow), (3) loss of NI and stable bidirectional CI (blue), (4) loss of both NI and bidirectional CI (white). The dotted and solid lines indicate the critical migration rates of NI and bidirectional CI, respectively. Bidirectional CI is stable if both *Wolbachia* strains stably coexist. Parameters: *l*_NI_ = 1, *h* = 0, *s* = 0.1. Under these parameter values of strong recessive NI, presence of NI has little effect on CI stability, whereas CI level strongly effects stability of nuclear divergence. *Graph B: Variable Dominance NI.* Critical migration rates for nuclear divergence as functions of the CI level for *h* = 1 (triangles), *h* = 0.99 (circles), *h* = 0.5 (boxes) *h* = 0 (diamonds). Other parameters: *l*_NI_ = 1, *s* = 0.1. Whereas CI has no effect when lethality dominance is 1 (as expected), it has a large effect when even a low level of F1 hybrid survival occurs (*h* =  0.99).

In general, the effect of bidirectional CI on nuclear incompatibilities is much stronger than for the unidirectional CI scenarios because bidirectional CI reduces gene flow more strongly. As seen in Figure 6, the effect on critical migration rates can be substantial, even for weak CI levels. For higher or even perfect CI, the stabilizing effect is much stronger and nuclear incompatibilities can be maintained even at high migration rates (fig. 6A). For example, 80% CI can result in an elevated critical migration rate of 8.9% (compared to 1.3% without *Wolbachia*). If CI is perfect, no hybrids survive and the genotypes are perfectly linked to one *Wolbachia* strain. In this case, critical migration rates are at least 17% (without local selection) or higher when local selection is included (19.6% for *s* = 0.1). In contrast, the stability of cytoplasmic divergence is less affected by nuclear incompatibilities, though some stabilization of CI occurs for weak CI (results not shown).

In summary, the results show that *Wolbachia* can have a strong stabilizing effect on nuclear hybrid zones under certain conditions. Surprisingly for cases of unidirectional CI, we show that infection polymorphisms can exist at higher migration rates when NI occurs in hybrids (*Scenario B, C*), and that nuclear and cytoplasmic incompatibility types complement each other, and can also act synergistically under some conditions (*Scenario C*).

### Analytical approximations

Above, we explained the effect of CI on the stability of nuclear divergence by *Wolbachia*-induced gene flow reduction. Here, we formalize the verbal arguments, and derive some analytical approximations. Gene flow modification by *Wolbachia* was analyzed previously for mainland-island models [26], [31]. It was shown that in comparison to no *Wolbachia* models (*Scenario A*), gene flow is reduced in *Wolbachia* scenarios. The gene flow reduction computes to

(1) 
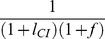
 for *Scenario B*,

(2) 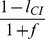 for *Scenario C*, and

(3) 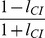 for *Scenario D*.

Approximations for the critical migration rates can be derived as follows. For a certain parameter set, let *m*_c,NI_(0) denote the critical migration rate for nuclear divergence in the absence of *Wolbachia* (*Scenario A*). Though we were not able to derive analytical approximations in the absence of *Wolbachia* (these need to be calculated numerically), we can use the numerically calculated values to get approximations for the *Wolbachia* scenarios. Let *m*_c,NI_(*l*_CI_) denote the critical migration rate for nuclear divergence in the presence of cytoplasmic incompatibility, where *l*_CI_ is the strength of CI. Dividing *m*_c,NI_(0) by the gene flow factors of the respective scenario yields good approximations for *m*_c,NI_(*l*_CI_):

(4) 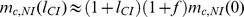 for *Scenario B*,

(5) 
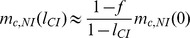
 for *Scenario C*, and

(6) 
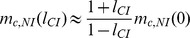
 for *Scenario D*.

**Figure 7:**
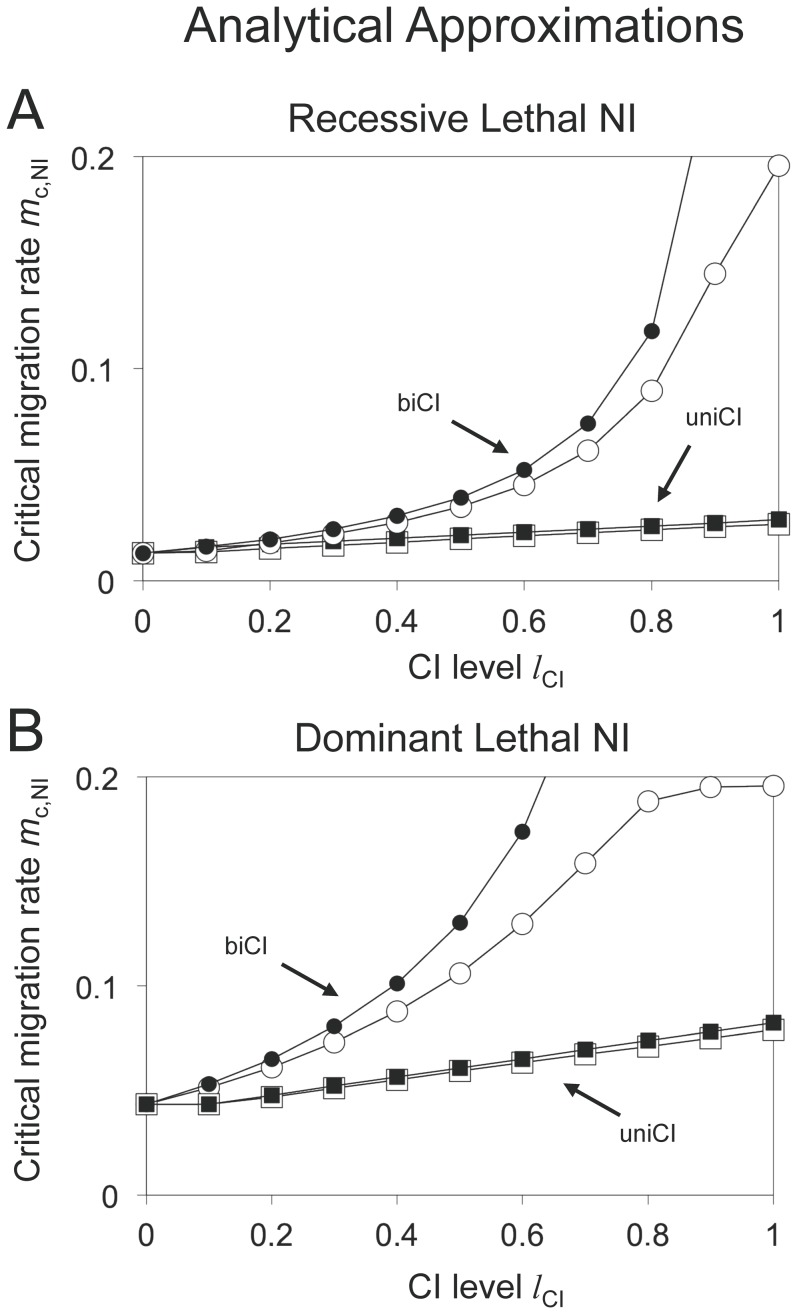
Analytical approximations of critical migration rates. Squares indicate *Scenarios B* (unidirectional CI with infected island population), and circles *Scenario D* (bidirectional CI). Black and white symbols represent numerical results and analytical approximations using formula (1), respectively. *Graph A: Recessive Lethal NI.* Parameters: *l*_NI_ = 1, *h* = 0, *s* = 0.1. *Graph B: Dominant Lethal NI.* Parameters: *h* = 0.99, *l*_NI_ = 1, *s* = 0.1. Approximations are especially good for unidirectional CI and low to moderate bidirectional CI.

Figure 7 shows the accuracy of the approximations for *Scenario B* and *D*. These are especially good for unidirectional CI and moderate bidirectional CI.

## Discussion

In this study, we investigated the effect of *Wolbachia* on the stability of hybrid zones formed after the Dobzhansky-Muller model. We considered three *Wolbachia* scenarios in detail that involve either uni- or bidirectional CI (*Scenarios B*–*D*). Our main result is that *Wolbachia*-induced CI favors maintenance of nuclear incompatibilities in the face of migration over a broad range of conditions. In general, the effect is strongest for recessive nuclear incompatibilities and strong uni- or bidirectional CI. An interesting special case occurred in the scenario with an infected mainland (*Scenario C*), where strong unidirectional CI and nearly dominant NI synergistically stabilize each other leading to high critical migration rates for both incompatibilities. These results support the view that *Wolbachia* could play an important role in maintaining divergence between populations, thus facilitating host speciation.

It is generally assumed that alleles causing incompatibilities are usually recessive in the early stages of reproductive isolation. This is supported by the observation that Haldane's rule arises in part due to exposure of recessive incompatibilities on the X chromosome (although they must interact with a dominant autosomal incompatibility), and that sterility and inviability are typically stronger among F_2_ hybrids, in which recessive incompatibilities are exposed. Theoretical analysis indicates that recessive incompatibilities hardly reduce gene flow between parapatric populations [3], [42]. Recessive alleles, only exposed to selection in the F_2_ hybrid and later generation, easily migrate, leading to a collapse of genetic divergence. A *Wolbachia* infection can therefore play an important role in maintaining the nuclear incompatibilities, especially when CI levels are high. CI levels have been found to cover a wide range, from fairly weak CI in some *Drosophila* [43] to complete CI in *Nasonia* [15]. Our model suggests that cytoplasmic incompatibility can increase the stability of genetic divergence substantially over a broad range of parameters (fig. 4–6). Particularly large effects occur for strong bidirectional CI and recessive Dobzhansky Muller incompatibilities. However, large effects are also found for unidirectional CI, where *Wolbachia* and nuclear incompatibilities can be mutually reinforcing. The only case where CI had no effect was for full NI level and complete dominance. The reinforcement of genetic incompatibilities by *Wolbachia*-induced CI could therefore be a common phenomenon.

While there is both empirical and theoretical evidence that bidirectional CI might promote speciation [15], [25], it has been argued that it occurs too rarely to be considered an important factor in the evolution of insects (or arthropods) [29]. Since bidirectional CI requires at least two populations of one species to harbor infections of two different strains, bidirectional CI between incipient species was expected to occur infrequently. Assessments of the expected rarity of bidirectional CI were based on earlier estimates of *Wolbachia* infection frequencies of around 20% [18]. Given 20% infected species, the “random” expectation of two incipient species infected with different *Wolbachia* would be 4%. In contrast, newer estimates of *Wolbachia* infection frequencies range from 40–70% [19], [20], yielding “random” expectations of 16% to 49% bidirectional situations. Furthermore, there are a number of documented bidirectional CI scenarios in insects. Examples include *Nasonia* [15], *Drosophila simulans* [44], the beetle *Chelymorpha alternans* [45], and several mosquito species [46], [47], although some of these may be the result of *Wolbachia* nuclear interactions.

In *Nasonia*, Breeuwer and Werren [15] showed that two sister species *Nasonia giraulti* and *N. vitripennis* produce progeny after being cured from infection but do not do so without antibiotic treatment, thus classifying *Wolbachia* infection as a significant isolating mechanism, at least under laboratory conditions. Offspring from cured individuals showed hybrid breakdown due to nuclear factors in the F_2_ generation. This could match our model with recessive incompatibilities. Bordenstein *et al.* [17] showed that bidirectional incompatibility also occurred in other species pairs, and the *Wolbachia* incompatibilities occur early in the speciation process in this genus.

There is also empirical evidence implying that this scenario could apply beyond insect-species. Gotoh *et al.* [39] examined 25 populations of a spider mite species, five of which were infected by *Wolbachia*. Crossing experiments displayed various forms of hybrid breakdown, which were caused by nuclear and cytoplasmic incompatibilities. In some crosses nuclear and cytoplasmic factors might have acted simultaneously, though it is difficult to determine which role nuclear or cytoplasmic factors play in particular. This is a general problem often addressed in speciation theory. Especially considering *Wolbachia*-induced CI, it is argued that *Wolbachia* can only play a role if they predate nuclear incompatibilities. In *Nasonia* it was shown, however, that CI preceded other incompatibilities [17]. On the other hand, it is also likely that speciation processes are driven by multiple interacting factors [16], [21], [37], [38], [48]. Especially within the framework of the Dobzhansky-Muller model, populations can diverge genetically and become infected by *Wolbachia* in allopatry. During secondary contact, both would contribute to reproductive isolation, simultaneously and not successively.

Unidirectional CI will occur between diverging allopatric populations (that come into secondary contact) when the ancestral source population lacked *Wolbachia* and one population subsequently acquired the infection, or when the ancestor was infected and the allopatric population lost the *Wolbachia*. There is evidence supporting both processes in nature (e.g., [27], [37], [45], [49], [50]), and there is ample evidence of closely related species being infected with different *Wolbachia* or one having the bacterium and another lacking it [23].

Early theoretical analyses of population dynamics indicated that *Wolbachia* infection would spread in a population once introduced [51], and thus, the isolating mechanism induced by *Wolbachia* would rapidly get lost after few generations. However, empirical studies have shown that unidirectional CI exists, apparently stably, between parapatric populations in nature [27], [45], [52]. Theoretical work indicates that infection differences between parapatric populations can be stable under certain conditions, but critical migration rates are generally low [34]. However, we find that combining unidirectional CI with nuclear incompatibilities expands the conditions for stability of unidirectional CI, as well as those for maintaining NI between populations.

There is a rapidly growing set of detailed studies examining *Wolbachia* variation both within and between closely related species. The most powerful analyses combine data on *Wolbachia*, mitochondrial, and nuclear genetic variation. To interpret these studies, the codynamics of mitochondria and *Wolbachia* is taken into consideration. A strong association of mitochondrial haplotype variation with infection status is indicative of high fidelity vertical transmission of the bacteria within the species, because both *Wolbachia* and mitochondria are maternally transmitted. Uncoupling of mitochondrial and *Wolbachia* variation indicates either infectious or paternal transmission. Incomplete maternal transmission of *Wolbachia* also leads to some uncoupling of infection status and mitochondria, as infected haplotypes can get converted to uninfected haplotypes.

Most studies (but not all, e.g. [53], [54]) reveal strong associations between mitochondrial haplotypes and *Wolbachia* infection. Here we will briefly discuss some examples relevant to the modeling efforts presented in this paper. Using mitochondrial, nuclear and *Wolbachia* variation data, Raychoudhury *et al.* [50] found three different patterns of *Wolbachia* transmission within the Nasonia species complex – most commonly found was horizontal (infectious) acquisition from other insects outside the genus with subsequent high fidelity maternal transmission. One instance of codivergence of *Wolbachia* during host speciation was detected, as well as one instance of introgression of *Wolbachia* and associated mitochondrial haplotypes across species boundaries. The latter finding is relevant to our models, and represents an example where hybridization between the species led to movement of *Wolbachia* across the species boundary, and subsequent spread of the *Wolbachia*/mitochondria combination, likely due to CI. There is no evidence of associated nuclear gene introgression between the species pair, suggesting that hybridization between the species was a rare event, which led to *Wolbachia* exchange but not significant nuclear exchange. Other possible examples of *Wolbachia* mitochondrial introgressions between species include the fig wasp *Ceratoloslen solmsi* [55], oak gall wasps [56], and Altica leaf beetles [57]. Other studies show divergent mitochondria haplotypes within a species that are associated with *Wolbachia* infection status [58]–[60]. These may represent hybridization events where the source has not yet been identified, or horizontal acquisition of *Wolbachia* from another taxon followed by high fidelity maternal inheritance and maintenance of an infection polymorphism for sufficient time for mitochondrial variation to accumulate.

As predicted by our model, introgressions of *Wolbachia* between hybridizing species or subspecies is expected to be a common outcome, and whether genetic and *Wolbachia* differences between the populations is maintained depends upon effective migration rates and fitnesses hybrid genotypes. Bella *et al*. [61] describe a hybrid zone occurring in the Pyrenees between two races of meadow grasshopper. There are two *Wolbachia* types present, which differ in frequencies between northern southern populations, suggesting incomplete introgression, although whether *Wolbachia* are playing a role in disrupting gene flow across the hybrid zone remains unclear. Further studies of the *Wolbachia*-mitochondrial associations across hybrid zones and possible *Wolbachia* effects on gene flow are needed.

In the present study, we have investigated the effect of *Wolbachia* on one pair of DMI loci. The analysis suggests that CI is a more efficient isolating barrier than DMI because CI is less likely to collapse for a broad range of parameters. It is argued that many incompatible pairs, which all have small effects, could play an important role in speciation [8]. In such a situation nuclear incompatibilities might be more stable than CI, especially if local selection is strong and/or fecundity reductions of *Wolbachia* are weak. This could result in a stabilizing effect of NI on CI for a broad parameter region. A further interesting question is, under which conditions will synergistic effects between NI loci and CI occur? One possible outcome could be that strong unidirectional CI is stabilized by multiple recessive DMI loci in a similar way as observed in *Scenario C*. This is because gene flow reduction by many weak nuclear incompatibilities might be sufficiently large to prevent the spread of *Wolbachia* effectively. The scenario will be further complicated by linkage relationships among different incompatibility loci, and addition of sex chromosomes, which have different association disequilibria with mitochondrial and *Wolbachia* in female versus male heterogametic species. Such an analysis is beyond the scope of this paper, and left for future studies.

*Wolbachia* also have a multitude of other effects on hosts that can contribute to genetic divergence and possibly promote speciation. For example, *Wolbachia* can influence mating behavior (e.g. [62], [63]), germline and ovarian development [63], [64], and resistance to viruses and parasites [65]–[67], and many of these effects likely involve co-evolutionary interactions between the endosymbiont and host which could contribute to hybrid breakdown among closely related species. In an interesting recent paper, Brucker *et al.* (2013) found that differences in gut microbiota between Nasonia species, and that regulation of these is disrupted in hybrids, contributing to hybrid mortality [68]. This principle could readily apply to *Wolbachia* regulation as well, with more rapid evolution of host genes involved in modulation of *Wolbachia* leading to disregulation in hybrids, for instance as found by Chafee *et al.* [69]. There is ample evidence for host genetic effects on *Wolbachia* phenotypes (e.g. [47], [70], [71]), many of which may be due to selection for suppression of *Wolbachia* [40], [72]. These could contribute to hybrid incompatibilities.

In summary, we find that *Wolbachia*-induced CI can stabilize nuclear genetic divergence in parapatric host populations under some general conditions. Due to the high abundance of *Wolbachia* in nature and given that not only bi-, but also unidirectional CI is influential in maintaining nuclear incompatibilities, our results are consistent with the idea that *Wolbachia* may play a general role in arthropod speciation processes.

## Supporting Information

Appendix S1 **Mathematical Model Description.**
(DOC)
