# Supplementary material for: Dobzhansky-Muller and Wolbachia-Induced Incompatibilities in a Diploid Genetic System
Source: PLoS One. 2014 Apr 23;9(4):e95488. doi: 10.1371/journal.pone.0095488 (PMC3997523; doi:10.1371/journal.pone.0095488)
Supplement: Appendix S1 — Mathematical Model Description. (DOC) [file pone.0095488.s001.doc]

**Appendix: Mathematical Model Description**

We consider a Dobzhansky-Muller model where one ancestral population splits into two temporarily isolated populations. During separation, alterations of populations' geno- and cytotypes can take place. The model describes the temporal dynamics after secondary contact of the geno-cytotype frequencies. It consists of a system of coupled difference equations that describe frequency changes in discrete time. Within each time step, the model describes the life cycle of individuals, which consists of three steps: migration, local selection and reproduction. The latter includes inheritance of nuclear alleles, cytoplasmic transmission of *Wolbachia*, and hybrid incompatibilities due to genetic and cytoplasmic factors. Individuals are diploid at all stages of life cycle, and generations are discrete and non-overlapping.

Frequencies of the different geno-cytotypes in subsequent generations are denoted by $p_{\vec{i}}$ and $p'_{\vec{i}}$ for the island and by $q_{\vec{i}}$ and $q'_{\vec{i}}$ for the mainland population. Thereby, $\vec{i}=(i_{1},i_{2},i_{3})$ is a 3-dimensional vector describing the different geno-cytotypes. The *A*-locus is characterized by *i*_1_ and the *B*-locus by *i*_2_. It holds that

$i_{1}=\left\{\begin{matrix} 0 \\ 1 \\ 2 \end{matrix}\begin{matrix} for \\ for \\ for \end{matrix}\begin{matrix} A_{m}A_{m} \\ A_{i}A_{m} \\ A_{i}A_{i} \end{matrix}\right.$, and (1)

$i_{2}=\left\{\begin{matrix} 0 \\ 1 \\ 2 \end{matrix}\begin{matrix} for \\ for \\ for \end{matrix}\begin{matrix} B_{m}B_{m} \\ B_{i}B_{m} \\ B_{i}B_{i} \end{matrix}\right.$. (2)

*Wolbachia* infection status is described by *i*_3_. For unidirectional CI, it holds that

$i_{3}=\left\{\begin{matrix} 1 \\ 0 \end{matrix}\begin{matrix} for \\ for \end{matrix}\begin{matrix} Wolbachia \\ No-Wolbachia \end{matrix}\right.$, (3)

and for bidirectional CI that

$i_{3}=\left\{\begin{matrix} 1 \\ 0 \end{matrix}\begin{matrix} for \\ for \end{matrix}\begin{matrix} W_{i} \\ W_{m} \end{matrix}\right.$. (4)

Individuals’ lifecycle starts with migration. A fraction *m* of island individuals is replaced by immigrants from the mainland. Frequencies on the island after immigration are denoted by and compute to

$p_{\vec{i}}^{+}=(1-m)p_{\vec{i}}+mq_{\vec{i}}$. (5)

After migration local viability selection takes place. In the island population allele *A_i_* is positively selected. Individuals with genotype *A_i_A_m_* and *A_i_A_i_* have a 1+*s*/2 respectively 1+*s* times higher probability to survive than *A_m_A_m_* individuals. Frequencies after local viability selection are denoted by $p_{\vec{i}}^{++}$ and compute to

$p_{\vec{i}}^{++}=\frac{1+i_{2}s}{\sum_{\vec{r}} (1+r_{2}s)p_{\vec{r}}^{+}}p_{\vec{i}}^{+}$. (6)

Third, individuals mate randomly and reproduction takes place. In order to formalize this last step of the life cycle, several weighting factors are defined that describe nuclear and cytoplasmic incompatibilities as well as inheritance of alleles and cytoplasmic elements.

Nuclear incompatibilities occur when both alleles *A*_i_ and *B*_m_ are present in a single individual. Thereby, the number of incompatible alleles and the dominance level *h* determine which proportion *l*_NI_ of progeny is inviable. This is described by weighting factors $N_{i_{1},i_{2}}$, which are defined as

$N_{i_{1},i_{2}}=\left\{\begin{matrix} 1 \\ 1-l_{NI} \\ 1-hl_{NI} \end{matrix}\right.\begin{matrix} for \\ for \\ else \end{matrix}\begin{matrix} i_{1}=0\vee i_{2}=2 \\ i_{1}=2\wedge i_{1}=0 \\ \end{matrix}$. (7)

Regarding the inheritance of alleles, we assume full recombination. An individual inherits one allele at each locus from one parent. If the parent is heterozygotic, each allele is transmitted to 50% of progeny. Assuming that *i* denotes the offspring’s genotype and *r* and *k* denote the genotype of parents, this is formalized by

$I_{r,k,i}=\left\{\begin{matrix} 1 & for & \left(r=k=i\neq1\right)\vee\left(i=1\neq r\neq k\wedge r+k=2\right) & \\ 0.5 & for & \left(k=1\vee l=1\right)\wedge\left(i=k\vee i=l\right) & \\ 0.25 & for & r=k=1\neq i & \\ 0 & else & & \end{matrix}\right.$, (8)

and holds for inheritance of alleles at both loci.

*Wolbachia* and cytoplasmic incompatibility is described by three weighting factors: one for *Wolbachia* transmission (*T*), one for fecundity costs of infection (*F*), and one for uni- and bidirectional CI (*L*). *Wolbachia* transmission is assumed to be strictly maternally. The corresponding weighting factors are

$T_{k_{3},i_{3}}=\left\{\begin{matrix} 1 & for & k_{3}=i_{3}=1 \\ 0 & else & \end{matrix}\right.$, (9)

where *k*_3_ and *i*_3_ denote the offspring’s and mother’s infection status, respectively. In the unidirectional CI scenario, *Wolbachia*-infected females are supposed to have (1-*f*) percent less offspring than uninfected females. This is described by

$F_{k}=\left\{\begin{matrix} 1 & for & k_{3}=0 \\ 1-f & for & k_{3}=1 \end{matrix}\right.$. (10)

Note that in the bidirectional CI scenario, all females are infected. Therefore, no relative reduction in fecundity is considered.

Cytoplasmic incompatibility occurs if the father is infected with a certain strain of *Wolbachia*, which is not present in the zygote. In this case, the fraction *l*_CI_ of offspring dies. This is modeled by

$L_{r_{3},i_{3}}=\left\{\begin{matrix} 1-l_{CI} & for & k_{3}=1\neq i_{3} \\ 1 & else & \end{matrix}\right.$, (11)

where *k*_3_ represents the father’s infection state, and *i*_3_ the infection state of an egg.

With these weighting factors we are able to write down the third and final step of the model. The geno-cytotype frequencies in the next generation are denoted by $p'_{\vec{i}}$ and compute to

$Wp'_{\vec{i}}=\sum_{\vec{k},\vec{r}} p_{\vec{k}}^{++}p_{\vec{r}}^{++}I_{k_{1},r_{1},i_{1}}I_{k_{2},r_{2},i_{2}}N_{i_{1},i_{2}}T_{r_{3},i_{3}}L_{k_{3},i_{3}}F_{r_{3}}$. (12)

Here *W* is the average fitness that compute to

$W=\sum_{\vec{i}} \sum_{\vec{k},\vec{r}} p_{\vec{k}}^{++}p_{\vec{r}}^{++}I_{k_{1},r_{1},i_{1}}I_{k_{2},r_{2},i_{2}}N_{i_{1},i_{2}}T_{r_{3},i_{3}}L_{k_{3},i_{3}}F_{r_{3}}$. (13)
